# 29-mRNA host response signatures for classification of bacterial infection, viral infection and disease progression in COVID-19 pneumonia: a post hoc analysis of the SAVE-MORE randomized clinical trial

**DOI:** 10.1186/s40635-025-00777-1

**Published:** 2025-06-30

**Authors:** Evdoxia Kyriazopoulou, Antigone Kotsaki, Asimina Safarika, Garyfallia Poulakou, Haralampos Milionis, Simeon Metallidis, Georgios Adamis, Archontoula Fragkou, Aggeliki Rapti, Pierluigi Del Vecchio, Ioannis Kalomenidis, Danae Kitzoglou, Andrea Angheben, Ilias Kainis, Konstantina Iliopoulou, Francesco Saverio Serino, Petros Bakakos, Vassiliki Tzavara, Sofia Ioannou, Lorenzo Dagna, Katerina Dimakou, Glykeria Tzatzagou, Maria Chini, Matteo Bassetti, Vasileios Kotsis, George Tsoukalas, Carlo Selmi, Sofia Nikolakopoulou, Michael Samarkos, Michael Doumas, Aikaterini Masgala, Ilias Papanikolaou, Aikaterini Argyraki, Karolina Akinosoglou, Styliani Symbardi, Periklis Panagopoulos, George N. Dalekos, Oliver Liesenfeld, Timothy E. Sweeney, Purvesh Khatri, Evangelos J. Giamarellos-Bourboulis

**Affiliations:** 1 4th Department of Internal Medicine, Medical School, National and Kapodistrian University of Athens, Athens, Greece; 2 3rd Department of Internal Medicine, Medical School, National and Kapodistrian University of Athens, Athens, Greece; 3 1st Department of Internal Medicine, Medical School, University of Ioannina, Ioannina, Greece; 4 1st Department of Internal Medicine, Medical School, Aristotle University of Thessaloniki, Thessaloniki, Greece; 5 1st Department of Internal Medicine, G. Gennimatas General Hospital of Athens, Athens, Greece; 6 Department of Internal Medicine, Elpis General Hospital, Athens, Greece; 7 2nd Department of Pulmonary Medicine, Sotiria General Hospital of Chest Diseases, Athens, Greece; 8 Unità Operativa Complessa (UOC) Malattie Infettive-Dipartimento Di Scienze Mediche E Chirurgiche, Fondazione Policlinico Gemelli IRCCS, Roma, Italy; 9 1st Department of Critical Care and Pulmonary Medicine, Medical School, Evangelismos General Hospital, National and Kapodistrian University of Athens, Athens, Greece; 10 10th Department of Internal Medicine, Tzaneio General Hospital of Piraeus, Athens, Greece; 11 Department of Infectious–Tropical Diseases and Microbiology, IRCSS Sacro Cuore Hospital, Negrar, Verona, Italy; 12 10th Department of Pulmonary Medicine, Sotiria General Hospital of Chest Diseases of Athens, Athens, Greece; 13 2nd Department of Internal Medicine, Thriasio General Hospital of Eleusis, Athens, Greece; 14 Department of Internal Medicine, Hospital of Jesolo, Lido Di Jesolo, Italy; 15 1st Department of Chest Medicine, Medical School, National and Kapodistrian University of Athens, Athens, Greece; 16 1st Department of Internal Medicine, Korgialeneion-Benakeion General Hospital, Athens, Greece; 17 Department of Therapeutics, Medical School, National and Kapodistrian University of Athens, Athens, Greece; 18 Unit of Immunology, Rheumatology, Allergy and Rare Diseases (UnIRAR), IRCCS Ospedale San Raffaele & Vita-Salute San Raffaele University, Milan, Italy; 19 5th Department of Pulmonary Medicine, Sotiria General Hospital of Chest Diseases, Athens, Greece; 20 1st Department of Internal Medicine, Papageorgiou General Hospital of Thessaloniki, Thessaloniki, Greece; 21 3rd Department of Internal Medicine and Infectious Diseases Unit, Korgialeneion-Benakeion General Hospital, Athens, Greece; 22 Infectious Diseases Clinic, Ospedale Policlinico San Martino IRCCS and Department of Health Sciences, University of Genova, Genoa, Italy; 23 3rd Department of Internal Medicine, Medical School, Aristotle University of Thessaloniki, Thessaloniki, Greece; 24 4th Department of Pulmonary Medicine, Sotiria General Hospital of Chest Diseases, Athens, Greece; 25 Humanitas Research Hospital, Milan, Italy; 26 Covid-19 Department, Asklipieio General Hospital of Voula, Athens, Greece; 27 1st Department of Internal Medicine, Medical School, National and Kapodistrian University of Athens, Athens, Greece; 28 2nd Department of Propedeutic Medicine, Medical School, Aristotle University of Thessaloniki, Thessaloniki, Greece; 29 2nd Department of Internal Medicine, Konstantopouleio General Hospital, Athens, Greece; 30 Department of Pulmonary Medicine, General Hospital of Kerkyra, Corfu, Greece; 31 Department of Internal Medicine, Sotiria General Hospital of Chest Diseases, Athens, Greece; 32 Department of Internal Medicine, University of Patras, Rion, Greece; 33 1st Department of Internal Medicine, Thriasio General Hospital of Eleusis, Athens, Greece; 34 2nd Department of Internal Medicine, Medical School, Democritus University of Thrace, 681 00 Alexandroupolis, Greece; 35 Department of Medicine and Research Laboratory of Internal Medicine, National Expertise Center of Greece in Autoimmune Liver Diseases, General University Hospital of Larissa, 41110 Larissa, Greece; 36 Inflammatix, Inc., Sunnyvale, CA USA; 37 Institute for Immunity, Transplantation and Infection, School of Medicine, Stanford University, Stanford, CA USA; 38 Center for Biomedical Informatics Research, Department of Medicine, Stanford University, Stanford, CA USA; 39 Hellenic Institute for the Study of Sepsis, Athens, Greece; 40 4th Department of Internal Medicine, ATTIKON University Hospital, 1 Rimini Street, 124 62 Athens, Greece

**Keywords:** COVID-19, Anakinra, Secondary infection, Bacterial infection, Viral infection, Severity, Mortality, Host response, Diagnostics, Gene expression

## Abstract

**Background:**

Biomarkers based on host response signatures are currently under development for the critically ill. We applied a 29-mRNA classifier for the diagnosis and prognosis of suspected acute infection and sepsis (TriVerity^™^, Inflammatix Inc.) in patients hospitalized with COVID-19.

**Methods:**

We applied three scores from locked classifiers (IMX-BVN-4 and IMX-SEV-4) from the 29-mRNA TriVerity^™^ blood test in participants of the SAVE-MORE randomized clinical trial (ClinicalTrials.gov NCT04680949) at baseline and days 4 and 7 of treatment, to classify bacterial infection, viral infection and decompensation. Participants were adults hospitalized with confirmed COVID-19 pneumonia and plasma soluble urokinase plasminogen activator receptor (suPAR) levels of ≥ 6 ng/ml, randomized to placebo or anakinra treatment.

**Results:**

A total of 471 patients were studied. At baseline nearly 90% had a Very Low or Low IMX-BVN-4 Bacterial Score and Moderate, High or Very High IMX-BVN-4 Viral Score. Anakinra treatment had an effect on the expression of genes indicating IMX-SEV-4 High or Very High scores after a 7 day treatment compared to baseline (12.9% of anakinra-treated patients continued being classified as high severity vs 20.4% of placebo-treated patients, p 0.046).

**Conclusions:**

The classifiers were well tested in COVID-19 pneumonia and may become a useful tool for hospitalized patients.

**Supplementary Information:**

The online version contains supplementary material available at 10.1186/s40635-025-00777-1.

## Background

The SARS-CoV-2 pandemic taught us that many patients meet the sepsis criteria [1, 2] implying that viral sepsis needs to be detected early. Early detection of viral infection may lead to avoidance of unnecessary use of antibiotics and early prediction of patients who will deteriorate, so that they become eligible for transfer to an intensive care environment. As symptoms at the start of illness may be subtle, various biomarkers have been investigated to diagnose sepsis and predict unfavorable outcomes [3].

The TriVerity^™^ Test (Inflammatix, Inc., Sunnyvale, CA) is a host-response-based diagnostic and prognostic test that quantifies the expression of 29 messenger RNAs (mRNAs) from whole blood to diagnose the presence of infection (bacterial, viral, or not infected) and predict illness severity (use of mechanical ventilation, vasopressors, and/or renal replacement therapy within 7 days) [4]. It has been developed and validated mainly in patients admitted to Emergency Departments (ED) with a suspicion of bacterial sepsis, but further validation in a wider population of critically ill patients, such as surgical or multi-injured, is ongoing [5, 6]. TriVerity has also been investigated in patients with COVID-19 and potential bacterial coinfections [7, 8]. The TriVerity Test is performed on the proprietary Myrna Instrument (Inflammatix, Inc.) with a turn-around time of approximately 30 min.

SAVE-MORE is a pivotal randomized clinical trial of anakinra, a recombinant human interleukin (IL)-1 receptor antagonist, in COVID-19 pneumonia [9]. Enrolled participants with early activation of the IL-1 cascade selected by increased blood concentrations of the biomarker suPAR (soluble urokinase plasminogen activator receptor) were randomized to treatment with placebo or anakinra. The trial showed a clinical benefit with anakinra treatment compared to placebo by day 28, as expressed by the World Health Organization (WHO) Clinical Progression Scale (WHO-CPS), and led to anakinra registration approval for the management of COVID-19 pneumonia by the European Medicines Agency (EMA) and Emergency Use Authorization by the US Food and Drug Administration (FDA) [10–12]. We applied the 29-mRNA classifier, generated on the Nanostring nCounter^(R)^ platform, to SAVE-MORE participants in order to investigate how this tool classifies patients hospitalized with COVID-19 pneumonia.

## Methods

### Patients

The SAVE-MORE trial is a double-blind randomized clinical trial (NCT04680949), approved by the National Ethics Committee of Greece (approval 161/20) and by the Ethics Committee of the National Institute for Infectious Diseases Lazzaro Spallanzani, IRCCS, in Rome (1 February 2021) [9]. The original trial was conducted from 23 December 2020 to 31 March 2021. In this trial, patients with confirmed COVID-19 pneumonia at high risk to develop severe respiratory failure (SRF), as indicated by levels of the biomarker suPAR in plasma ≥ 6 ng/ml, were 1:2 randomly allocated to once daily subcutaneous treatment with either placebo or anakinra 100 mg for 10 days in addition to Standard-of-Care (SoC). Severe respiratory failure was defined as progression to a respiratory ratio below 150 requiring non-invasive or invasive mechanical ventilation. Dexamethasone, remdesivir, and anticoagulation were allowed in the SoC at the discretion of treating physicians; other anti-cytokine drugs like tocilizumab were not allowed. Main exclusion criteria were: non-invasive or invasive mechanical ventilation at baseline, stage IV malignancy, any do-not-resuscitate decision, ratio of partial oxygen pressure to fraction of inspired oxygen less than 150, severe hepatic failure, any primary immunodeficiency, neutropenia, oral or intravenous corticosteroids more than 0.4 mg/kg/day of equivalent prednisone in the last 15 days, any anti-cytokine biologic treatment in the last month, hemodialysis, and pregnancy or lactation. All patients or their legal representatives provided written informed consent before enrollment.

Full clinical information was recorded including demographics, severity scores, comorbidities, predisposing conditions, and 28-day outcome. Bacterial coinfections and superinfections were judged by treating physicians blinded to the results, and proven infections were recorded taking into consideration clinical assessment, microbiological results (including specimen culture results) and other laboratory investigations using predefined criteria (Supplement). However, data involving the timing of bacterial superinfection were unavailable, and classifier measurements did not coincide with time of superinfection diagnosis. As a consequence, this was only examined from a prognostic standpoint, and statistical parameters to evaluate the performance of the classifier were not possible. Whole blood was drawn in PAXgene^(R)^ Blood RNA tubes (Becton Dickinson) at three timepoints (at baseline before start of the study drug and days 4 and 7 of treatment), along with other standard laboratory parameters. In the current analysis, only participants providing at least a baseline sample are included.

### Laboratory procedures

PAXgene blood RNA samples were shipped to Inflammatix (Sunnyvale, CA, USA) and processed by technicians blinded to clinical information. RNA extraction from PAXgene Blood RNA was performed in batched mode using a standardized protocol on the QiaCube^®^ as previously described [13, 14]. RNA targets were counted using the NanoString nCounter^®^ SPRINT Profiler from 150 ng of isolated RNA; the expression of four housekeeping genes (*CDIPT, KPNA6, RREB1, YWHAB*) was normalized using geometric-mean gene normalization, i.e., for each sample, counts for all genes were multiplied by a sample-specific factor so that the geometric means of housekeeping gene counts become equal across all samples described [11]. Among the full gene list is *IL1R2* gene [17]. In the current study, the Bacterial Viral Non-infected (BVN)-4 and the Inflammatix Severity (IMX-SEV)-4 algorithms were applied (the same locked classifiers as the clinical version of the test, but applied to NanoString data) [15, 16]. The 4th generation classifiers have a few different genes (certain changes were necessary in transitioning to a rapid point-of-care platform) and more patients available in the training data. The training of the version 4 classifiers is described elsewhere [18]. The classifiers were applied blinded to clinical information. The scores are each separated into 5 bands (Very Low, Low, Moderate, High, Very High) according to preset thresholds.

### Statistical analysis

Categorical data were presented as frequencies and confidence intervals (CI); continuous variables with normal distribution as mean with standard deviation (SD). Fisher’s exact test was used for comparison of categorical data, whereas Student’s t-test/ANOVA or non-parametric Mann–Whitney/Kruskal–Wallis tests were used for the comparison of continuous data, as appropriate. McNemar’s test was used for paired comparisons of categorical data. Odds ratio (OR) with CI was calculated for categorical data. Ordinal regression analysis was used to compare likelihood for bacterial and viral infection at days 4 and 7 between the two arms of treatment. Logistic regression analysis was used to detect the impact of Severity score at baseline on clinical outcomes at day 28. Any two-sided *p* value < 0.05 was considered statistically significant. Statistical analysis was performed using the software SPSS version 29.0.

## Results

### Patients

In the original trial, 594 patients were enrolled; 189 were treated with SoC and placebo, and 405 were treated with SoC and anakinra. The original trial was conducted before vaccination was made available for the Greek population except for healthcare workers, so all enrolled patients were not vaccinated. The predominant variant of that period was delta. In the present analysis, 471 patients consenting to blood draw and providing at least a baseline blood sample were included, of which 145 patients were allocated to treatment with SoC and placebo and 326 patients to treatment with SoC and anakinra. Characteristics of anakinra- and placebo-treated patients are presented in Supplementary Table 1. Blood samples were available from 471 patients at baseline, 394 patients at day 4 of treatment, and 392 patients at day 7 of treatment.

### Classification of bacterial and viral infection

**Fig. 1:**
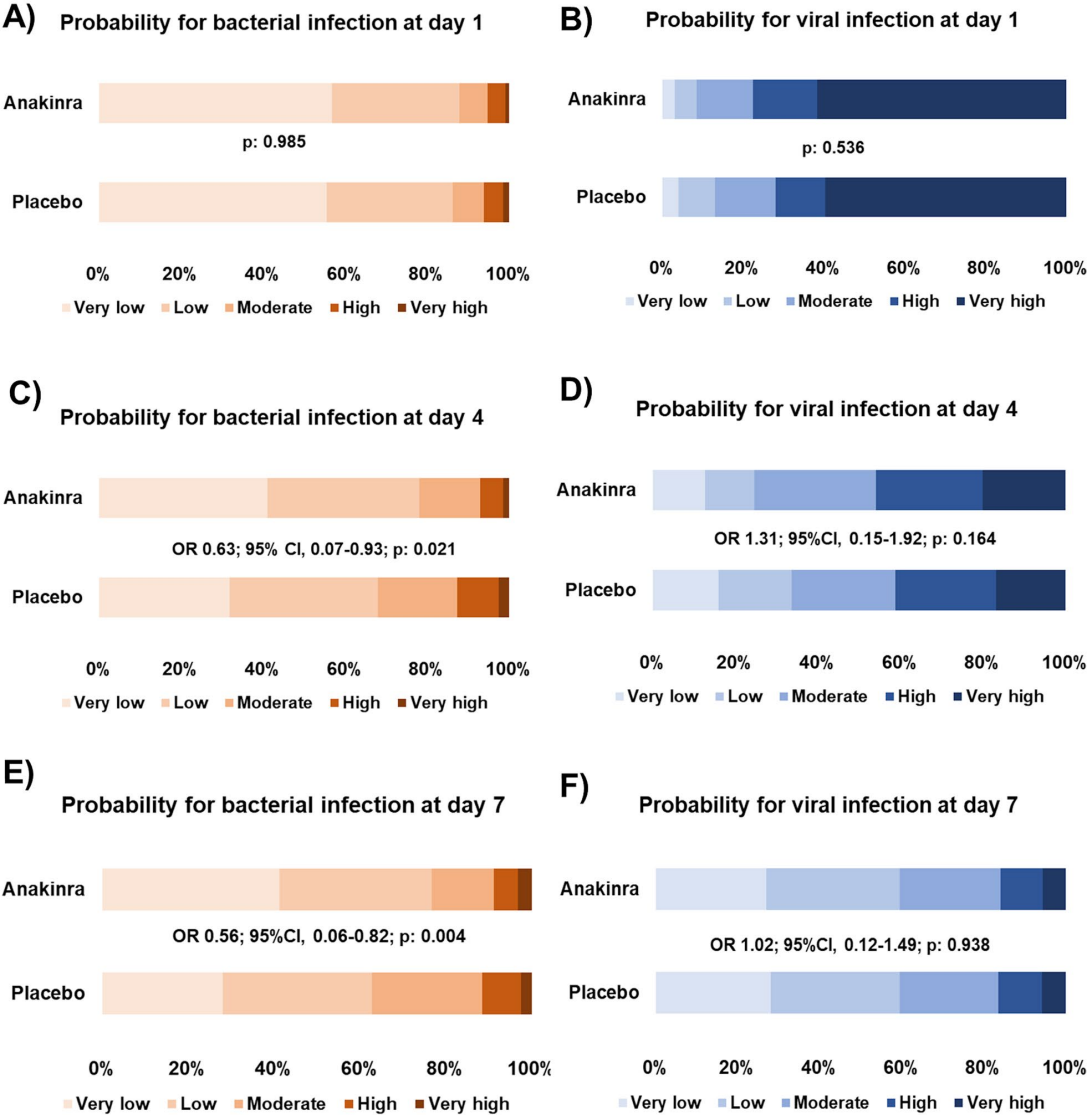
Classification of patients according to the likelihood of **A** bacterial infection at baseline; **B** viral infection at baseline; **C** bacterial infection at day 4; **D** viral infection at day 4; **E** bacterial infection at day 7; **F** viral infection at day 7. Arm of treatment (anakinra or placebo) is indicated in the left of each panel. p values of comparison between the two arms of treatment at baseline by the Chi-square test are provided. p values, odds ratios (OR) and 95% confidence intervals (CI) of ordinal regression analysis between the two arms of treatment at days 4 and 7 are provided

At baseline, patients of both arms of treatment had similar BVN-4 Bacterial Scores (88% Very Low or Low in anakinra- and 86.3% in placebo-treated patients; Fig. 1a) and BVN-4 Viral Scores (91.3% Moderate, High or Very High in anakinra- and 87% in placebo-treated patients; Fig. 1b). There were no differences between those classified as Very Low/Low Viral and Moderate/High/Very High Viral for severity of pneumonia (*p* 0.676), age (*p* 0.258) and Charlson’s comorbidity index (*p* 0.971). At day 4 of treatment, anakinra-treated patients had a decreased risk for bacterial infection (OR 0.63; 95% CI 0.07–0.93; p 0.021; Fig. 1c) compared to placebo-treated patients but shared a similar risk for viral infection (OR 1.31; 95%CI 0.15–1.92; *p* 0.164; Fig. 1d). The profile of the type of infection was shifted towards bacterial infection compared to baseline for both arms of treatment. More precisely, among anakinra-treated patients at day 4, BVN-4 Bacterial Scores were: 78.2% Very Low or Low, 14.9% Moderate, and 7% High or Very High compared to 88, 6.8 and 5.2% at baseline, respectively (p_McNemar_ < 0.001). Among placebo-treated patients at day 4, BVN-4 Bacterial Scores were: 68% Very Low or Low, 19.3% Moderate and 12.6% High or Very High compared to 86.3, 7.5 and 6.2% at baseline, respectively (p_McNemar_ < 0.001). This shift toward greater odds for bacterial infection remained at day 7 (p_McNemar_:0.004 for anakinra-treated patients and p_McNemar_ < 0.001 for placebo-treated patients, respectively, compared to baseline; Fig. 1e, f). The odds for bacterial infection was higher in placebo-treated patients compared to anakinra-treated patients (OR 0.56; 95%CI 0.06–0.82; *p* 0.004; Fig. 1e). This finding is consistent with increased infection risk in the placebo arm; 23 placebo-treated patients (15.9%) suffered from secondary bacterial infection compared to 30 (9.2%) anakinra-treated patients (p0.035).

### Classification of viral infection and association with viral load

The viral load in nasopharyngeal samples of the whole cohort decreased throughout the follow-up days 4 and 7 compared to baseline. Median (Q1–Q3) values of Ct of SARS CoV-2 polymerase chain reaction (PCR) *ORF* gene were 24.9 (20.4–29.3) at baseline; 29.0 (24.8–32.5) at day 4; and 31.2 (27.8–35) at day 7, respectively (p_Friedman_ < 0.001). The viral load correlated with the BVN-4 score for viral infection at all three timepoints; Spearman’s rho correlation coefficient was -0.399 for baseline (*p* < 0.001), − 0.247 for day 4 (*p* < 0.001), and −0.253 for day 7 (*p* < 0.001), respectively. Classification to viral infection was not associated with length of hospital stay (data not shown).

### Severity score at baseline and unfavorable outcome

**Fig. 2:**
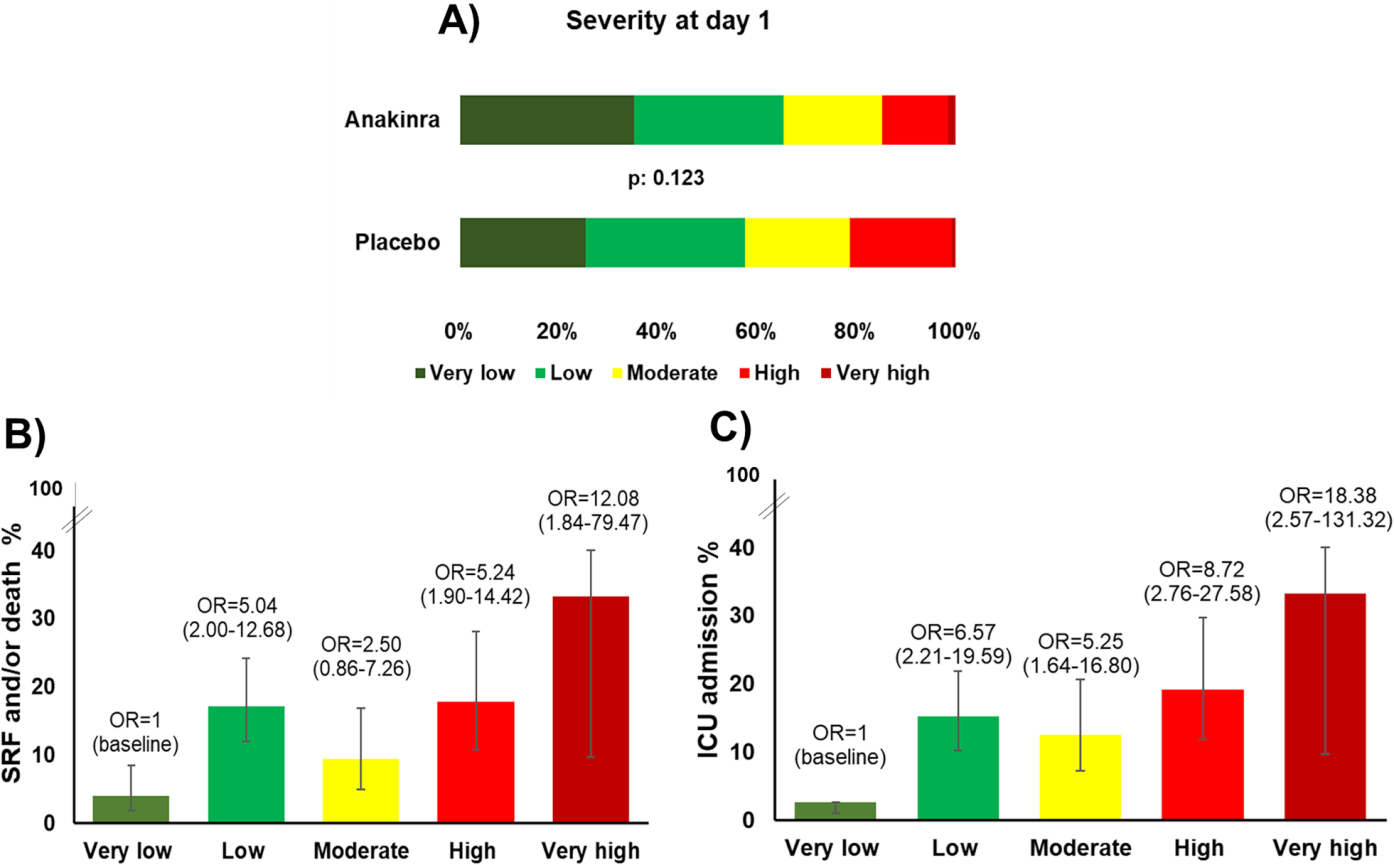
**A** Classification of patients according to the IMX-SEV-4 Severity Score at baseline, between anakinra- and placebo-treated patients. p value of comparison by the Chi-square test is provided. Proportions of patients of both arms who **B** developed severe respiratory failure (SRF) and/or died; and **C** required admission in intensive care unit (ICU) by day 28 according to the likelihood of severity at baseline. Odds ratios (OR) and 95% confidence intervals (CI) of categorical logistic regression analysis are provided

At baseline, patients in both arms of treatment had similar risk stratification in IMX-SEV-4 Severity Scores (Fig. 2a). Higher Severity Score at baseline was associated with development of SRF and higher need for Intensive Care Unit (ICU) admission within 28 days. More precisely, 55 patients developed SRF; of these, the SEV-4 Severity Scores at baseline were: 10.9% (6 patients) Very Low, 45.5% (25 patients) Low, 16.4% (9 patients) Moderate, 23.6% (13 patients) High, and 3.6% (2 patients) Very High. In the 416 patients who did not develop SRF, respective scores were found in 34.9, 28.8, 20.9, 14.4, and 1.0% (*p* < 0.001; Fig. 2b, Table 1). Fifty-four patients were admitted in the ICU; in these patients the SEV-4 Severity Scores at baseline were: 7.4% (4 patients) Very Low, 40.7% (22 patients) Low, 22.2% (12 patients) Moderate, 25.9% (14 patients) High, and 3.7% (3 patients) Very High. In the 417 patients who did not require ICU admission the respective scores were 35.3, 29.5, 20.1, 14.1 and 1.0% (*p* < 0.001; Fig. 2c). The baseline SEV-4 Severity Score correlated with other baseline clinical and laboratory variables which also define severity; Spearman’s rho correlation coefficient was 0.191 for Sequential Organ Failure Assessment (SOFA) score (*p* < 0.001), − 0.254 for PaO2/FiO2 (*p* < 0.001), 0.215 for C-reactive protein (CRP) (*p* < 0.001) and 0.125 for lactate (*p* 0.032), respectively.

**Table 1:** IMX-SEV-4 Severity score at baseline as independent predictor of outcome

Variable	Univariate analysis		Multivariate analysis	
	Odds ratio (95% CIs)	P	Odds ratio (95% CIs)	p
Age ≥ 65 years	1.40 (0.80–2.45)	0.247	–	
Male gender	2.87 (1.47–5.60)	0.002	2.80 (1.42–5.53)	0.003
Charlson’s comorbidity index > 2	1.35 (0.73–2.49)	0.344	–	
Severe pneumonia*	12.13 (1.65–89.06)	0.014	3.27 (0.15–73.15)	0.455
Intake of dexamethasone	11.94 (1.63–87.63)	0.015	3.45 (0.15–77.67)	0.436
IMX-SEV-4 score at baseline	1.44 (1.12–1.85)	0.004	1.30 (1.01–1.68)	0.049
Body mass index > 30	0.84 (0.47–1.52)	0.574	–	
Anakinra treatment	0.54 (0.30–0.95)	0.033	0.54 (0.30–0.98)	0.043

Univariate and multivariate forward conditional logistic regression analysis of progression into severe respiratory failure (SRF) and/ or death by day 28

CI: confidence interval; suPAR: soluble urokinase plasminogen activator receptor

^*^Defined as oxygen saturation less than 90% or more than 30 breaths/min or signs of respiratory distress

### Severity score at follow-up days 4 and 7 and unfavorable outcome

At day 4, the status of WHO-CPS (a scale from 0 to 10) was available for 471 patients; of these 2 were asymptomatic with positive PCR (WHO-CPS 1), 87 hospitalized without oxygen (WHO-CPS 4), 303 hospitalized with oxygen by mask or nasal prongs (WHO-CPS 5), 58 on high-flow oxygen therapy (WHO-CPS 6), 1 mechanically ventilated with respiratory ratio > 150 (WHO-CPS 7), 11 mechanically ventilated with respiratory ratio < 150 or in need of vasopressors (WHO-CPS 8) and 9 mechanically ventilated with respiratory ratio < 150 and in need of vasopressors and dialysis (WHO-CPS 9). The day-4 WHO-CPS status correlated with the day-4 TriVerity severity score (Spearman’s rho correlation coefficient 0.288, *p* < 0.001). At day 7, the status of WHO-CPS was available for 392 patients. One, 3, 154, 179, 33, 2, 17, and 3 patients had WHO-CPS scores 2, 4, 5, 6, 7, 8 and 9, respectively. The day-7 WHO-CPS status of the patients correlated with the day-7 TriVerity severity score (Spearman’s rho correlation coefficient 0.237, *p* < 0.001). Severity scores at days 4 and 7 of follow up were also associated with progression into SRF and ICU admission within 28 days (Supplementary Fig. 1).

### Effect of treatment on sequential IMX-SEV-4 severity score

Among 394 patients with serial measurements of the classifier, 42.9% were classified in the same risk band at day 4 compared to baseline, and 51.8% was classified in the same risk band at day 7 compared to day 4. At day 4, 21.3% patients were classified in a band of higher risk and 64.2% in a band of lower risk. At day 7, 25.4 and 22.8% was classified in a band of higher and lower risk, respectively.

**Fig. 3:**
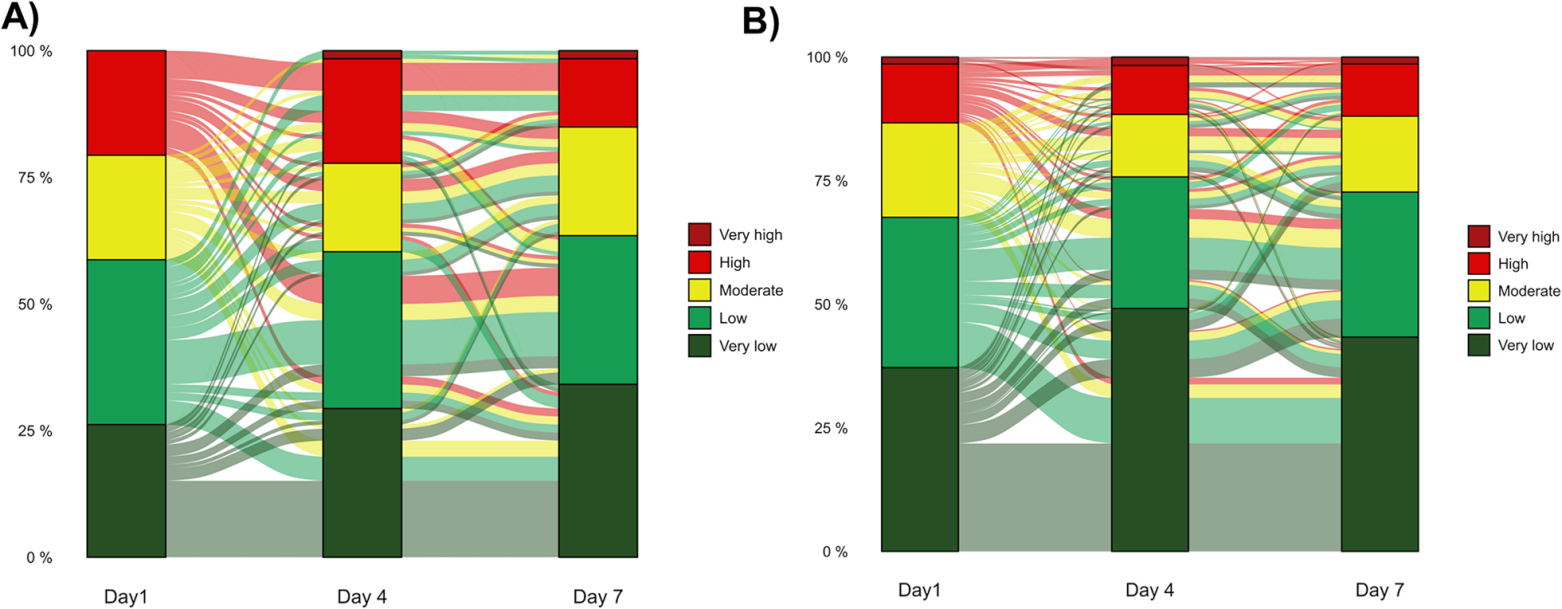
Alluvial plots of classification according to the IMX-SEV-4 Severity Score among SAVE-MORE participants treated with **A** standard-of-care (SoC) and placebo and **B** standard-of-care (SoC) and anakinra, from baseline to days 4 and 7 of treatment

Twenty-eight (20.4%) placebo-treated patients remained or turned stratified as High or Very High risk under a 7-day treatment compared to baseline (Fig. 3a), whereas only 40 (12.9%) anakinra-treated patients remained or turned stratified as High risk at day 4 compared to baseline (p_Chi-square_: 0.046; Fig. 3b). Fifteen patients (24.6%) of those who remained or turned stratified as High or Very High risk suffered a bacterial superinfection within 28 days of follow-up compared to only 25 (7.6%) who did not remain or turned stratified as High or Very High risk (*p* < 0.001).

## Discussion

In this post hoc analysis of the SAVE-MORE trial, we showed that the IMX BVN-4 classifier diagnosed patients with COVID-19 pneumonia as very likely to suffer from a viral infection. The IMX BVN-4 classifier results after 7 days shifted the classification of infection to a bacterial origin in some patients. Severity classification by the IMX SEV-4 classifier was associated with unfavorable outcome at day 28. A greater proportion of placebo-treated patients continued after 7 days to express genes indicating high risk in contrast to patients on anakinra treatment.

The TriVerity^™^Test is an innovative host-response assay combining the expression of 29 genes measured in peripheral blood to provide three independent readouts that reflect the likelihood of a bacterial infection, the likelihood of a viral infection, and the risk of severe illness, based on the need for critical care. The test, previously granted Breakthrough Device Designation by the FDA, was recently cleared by the FDA (January 2025). It has been developed and validated so far for use in ED in patients with suspicion of sepsis [13, 15, 16]; results of the FDA registrational study have recently been reported [19]. Proposed cutoffs provide sensitivity and specificity that exceed 95% for the diagnosis of bacterial sepsis [20]. Recently, the test also showed a good performance among patients with suspected influenza [21]. These results are consistent with those from Zheng et al. showing that host response to viral infections is conserved [22]. In our study, including only patients with confirmed viral pneumonia caused by the SARS-CoV-2 virus, classification by TriVerity viral results was considered successful; as it was expected, almost all patients were diagnosed very likely or likely to suffer from a viral illness. The lack of uninfected controls, however, did not allow calculation of sensitivity and specificity of the test’s performance.

A very interesting finding of our study is the shift to higher risk of infection of bacterial origin after 7 days from baseline evaluation. To the best of our knowledge, this is the first study evaluating the classifiers in serial samples of the same patients at three different timepoints. Published literature supports a low rate of bacterial co-infection among COVID-19 patients at baseline, but the rate of superinfection, especially when patients are admitted to the ICU, is higher [23, 24]. Our study corroborates with this finding; at baseline, a very low proportion of patients was likely to have bacterial infection. The higher likelihood after 7 days for a bacterial infection predicting the development of clinically documented secondary infections, underlines the test’s value in this setting. Secondary infections in COVID-19 patients are associated with higher mortality rates and early diagnosis is key to administer appropriate antibiotics [24]. Clinicians may suspect secondary infections by elevation of serum biomarkers, such as CRP and/or procalcitonin (PCT). In some cases, such as after administration of immunomodulation with dexamethasone and tocilizumab, kinetics of these biomarkers is slow or even suppressed [25, 26]; TriVerity may serve as an alternative diagnostic tool in such cases [27]. In our study, however, potential bias from immunotherapy with anakinra switching the host response cannot be eliminated and further research is needed.

COVID-19 pneumonia is highly heterogeneous ranging from asymptomatic disease to critical illness requiring admission in the ICU and mechanical ventilation representing viral sepsis [2]. To this end, critical COVID-19 may share similarities with other critical syndromes such as sepsis, pancreatitis and trauma, in which host response is key in pathophysiology and may be used to guide personalized treatment after development of appropriate biomarkers [28, 29]. In our study, we applied the classifiers in a different patient population from previously studied cohorts in an effort to detect the generalizability of this host-response biomarker in the critical care setting [30]. Our cohort consisted of hospitalized patients with COVID-19 pneumonia who met the criteria of sepsis by the SOFA score. The classifiers predicted patients who developed SRF and required ICU admission. Participants of the SAVE-MORE trial were already selected by plasma suPAR levels; values ≥ 6 g/ml predict unfavorable outcome at day 28 with a high sensitivity and specificity and the classification tool may have an additive value [31]. Another interesting novel finding of the current study is a small but important impact of anakinra treatment on host-response. Patients treated with anakinra were less likely to continue or turn to express high-risk genes when compared to placebo-treated patients.

Several limitations of the study need to be addressed: (a) the retrospective application of the classifiers in prospectively collected samples not allowing to evaluate application in real life; (b) the lack of samples from all patients at all three timepoints; (c) the application at a cohort where viral strains are different than those prevailing nowadays; (d) the lack of non-infected controls to calculate TriVerity’s performance (sensitivity, specificity, etc.) underlying the necessity of further research; (e) the lack of routine PCT measurements to compare classification of bacterial infections; (f) the study in patients with already suPAR ≥ 6 ng/ml which may not reflect the full spectrum of COVID-19 cases, and (g) the impact of anakinra immunomodulatory effects which may have changed test results independent of actual reference standard outcome.

## Conclusions

The IMX BVN-4 and SEV-4 classifiers, measured from a single blood draw, classified the risk of progression into SRF and of ICU admission among patients with COVID-19 pneumonia. More research is needed, including non-infected comparators to better understand their performance.

## Supplementary Information

Supplementary Material 1.

Supplementary Material 2.

## Data Availability

All data analyzed are presented in manuscript, tables and figures. Additional data are available from the corresponding author upon request.

## Abbreviations

AUC: Area under curve
BVN: Bacterial viral non-infected
CI: Confidence interval
CRP: C-reactive protein
ED: Emergency department
EMA: European Medicines Agency
FDA: Food and Drug Administration
ICU: Intensive care unit
IL: Interleukin
IMX-SEV: Inflammatix severity
mRNA: Messenger RNA
OR: Odds ratio
PCR: Polymerase chain reaction
PCT: Procalcitonin
ROC: Receiver operator characteristics
SD: Standard deviation
SOC: Standard-of-care
SOFA: Sequential Organ Failure Assessment
SRF: Severe respiratory failure
suPAR: Soluble urokinase plasminogen activator receptor
WHO-CPS: World Health Organization Clinical Progression Scale
