# Supplementary material for: 29-mRNA host response signatures for classification of bacterial infection, viral infection and disease progression in COVID-19 pneumonia: a post hoc analysis of the SAVE-MORE randomized clinical trial
Source: Intensive Care Med Exp. 2025 Jun 30;13:67. doi: 10.1186/s40635-025-00777-1 (PMC12206681; doi:10.1186/s40635-025-00777-1)
Supplement: Supplementary file 1 — Supplementary Material 1. [file 40635_2025_777_MOESM1_ESM.docx]

**Supplement**

**Definitions of secondary bacterial infections**

Bloodstream infection: Presence of at least one positive blood culture, yielding a pathogen that is not a skin commensal (e.g. coagulase-negative *Staplylococcus* spp).

Acute pyelonephritis: Presence of ≥10 leukocytes/high-power field in urine sediments with positive leukocyte esterase in urine and presence of at least two of the following:

- fever (tympanic or oral temperature ≥38^0^C; rectal temperature ≥38.3^0^C);
- dysuria, increased urinary frequency or urgency;
- flank pain or lumbar pain at palpation;
- consistent ultrasound findings.^1,2^

Community-acquired pneumonia (CAP): New or evolving infiltrate on chest X-ray with presence of at least two of the following:

- new onset or worsening of cough;
- dyspnea;
- auscultatory findings consistent with pulmonary consolidation

AND presence of at least one of the following:

- PCT ≥0.25 μg/liter;

hypoxemia pO_2_ ≤60mm Hg or oxygen saturation ≤90% in room air;

- respiratory rate ≥20 breaths/min.^3,4^

Healthcare-associated pneumonia (HCAP) or CAP with predisposing factors for multidrug resistant organisms: New or evolving infiltrate on chest X-ray in a patient on chronic dialysis or facilitated by residency in long-term care or by previous hospitalization in the last 3 months, with presence of at least two of the following:

- new onset or worsening of cough;
- dyspnea;
- auscultatory findings consistent with pulmonary consolidation

AND presence of at least one of the following:

- PCT ≥0.25 μg/liter;
- hypoxemia pO_2_ ≤60mm Hg or oxygen saturation ≤90% in room air;
- respiratory rate ≥20 breaths/min.^3,4^

Hospital-acquired pneumonia (HAP): New or evolving infiltrate on chest X-ray with onset >48 hours from hospital admission with presence of at least two of the following:

- new onset or worsening of cough or dyspnea;
- purulent tracheobronchial secretions;
- auscultatory findings consistent with pulmonary consolidation

AND presence of at least one of the following:

- PCT ≥0.25 μg/liter;
- hypoxemia pO_2_ ≤60mm Hg or oxygen saturation ≤90% in room air;
- respiratory rate ≥20 breaths/min.^3^

Ventilator-associated pneumonia (VAP): New or evolving infiltrate on chest X-ray with onset >48 hours from start of mechanical ventilation and presence of purulent tracheobronchial secretions, and auscultatory findings consistent with pulmonary consolidation

AND presence at least one of the following:

- PCT ≥0.25 μg/liter;
- clinical pulmonary infection score (CPIS) ≥6. CPIS is a clinical score based on body temperature, leukocyte count, volume and appearance of tracheal secretions, arterial oxygenation, chest radiograph findings, gram stain results, results of culture of tracheal aspirate specimens to predict the presence of VAP. Each parameter takes a score from 0 to 2.^5,6^

Biliary infection: Presence of

- Pain at the right upper quadrant
- Fever (tympanic or oral temperature ≥38^0^C, rectal ≥38.3^0^C)
- Consistent ultrasound or CT findings^7^

**Table 1. Characteristics of patients by group of treatment.**

|  | **Placebo + SoC (N=145)** | | **Anakinra + SoC (Ν=326)** | **p** |
| --- | --- | --- | --- | --- |
| Age, years, mean (SD) | 62 (11) | | 62 (12) | 0.840 |
| Male sex, n (%) | 84 (57.9) | | 190 (58.3) | >0.99 |
| Body mass index, mean (SD) | 30.2 (5.7) | | 29.6 (5.5) | 0.261 |
| Charlson’s comorbidity index, mean (SD) | 2.2 (1.5) | | 2.3 (1.6) | 0.534 |
| SOFA score, mean (SD) | 2.4 (1.1) | | 2.4 (1.1) | 0.925 |
| Severe pneumonia*, n (%) | 129 (89.0) | | 266 (81.6) | 0.057 |
| WHO Clinical Progression Scale, n (%) |  | |  | 0.464 |
| Hospitalized, no oxygen | 10 (6.9) | | 30 (9.2) |  |
| Hospitalized, oxygen by mask or nasal  prongs | 124 (85.5) | | 276 (84.7) |  |
| Hospitalized, high-flow oxygen therapy | 11 (7.6) | | 20 (6.1) |  |
| Comorbidities, n (%) |  | |  |  |
| Type 2 diabetes mellitus | 23 (15.9) | | 53 (16.3) | >0.99 |
| Chronic heart failure | 2 (1.4) | | 12 (3.7) | 0.244 |
| Chronic renal disease | 1 (0.7) | | 5 (1.5) | 0.672 |
| Chronic obstructive pulmonary disease | 7 (4.8) | | 13 (4.0) | 0.631 |
| SARS CoV-2 detection in the blood, n (%) | 36 (25.5) | | 72 (22.9) | 0.552 |
| SARS CoV-2 PCR *ORF 1ab* in the blood, Ct, median (Q1-Q3) | 40 (40-40) | | 40 (40-40) | 0.512 |
| SARS CoV-2 PCR *N* in the blood, Ct, median (Q1-Q3) | 40 (39.8-40) | | 40 (40-40) | 0.374 |
| Co-administered medications, n (%) |  |  |  |  |
| Remdesivir | 104 (71.7) | | 234 (71.8) | >0.99 |
| Dexamethasone | 128 (88.3) | | 268 (82.2) | 0.103 |
| Prophylactic low molecular weight heparin | 94 (97.9) | | 224 (99.1) | 0.585 |
| Outcomes, n (%) |  | |  |  |
| Incidence of SRF and/or death at day 28 | 47 (32.4) | | 67 (20.6) | 0.007 |
| ICU admission | 23 (15.9) | | 30 (9.2) | 0.040 |
| Secondary infection | 23 (15.9) | | 30 (9.2) | 0.040 |

*defined as oxygen saturation less than 90% or more than 30 breaths/min or signs of respiratory distress

Abbreviations: ICU, intensive care unit; SD, standard deviation; SoC, standard-of-care; SOFA, sequential organ failure assessment; SRF, severe respiratory failure.

**Supplementary Figure 1** Classification of patients according to the IMX-SEV-4 Severity Score a) at day 4, between patients who developed severe respiratory failure (SRF) and/or died; and those who did not, b) at day 4, between patients who required admission in intensive care unit (ICU) by day 28 and those who did not, c) at day 7, between patients who developed severe respiratory failure (SRF) and/or died; and those who did not, and d) at day 7, between patients who required admission in intensive care unit (ICU) by day 28 and those who did not. P-values of Chi-square test are provided.


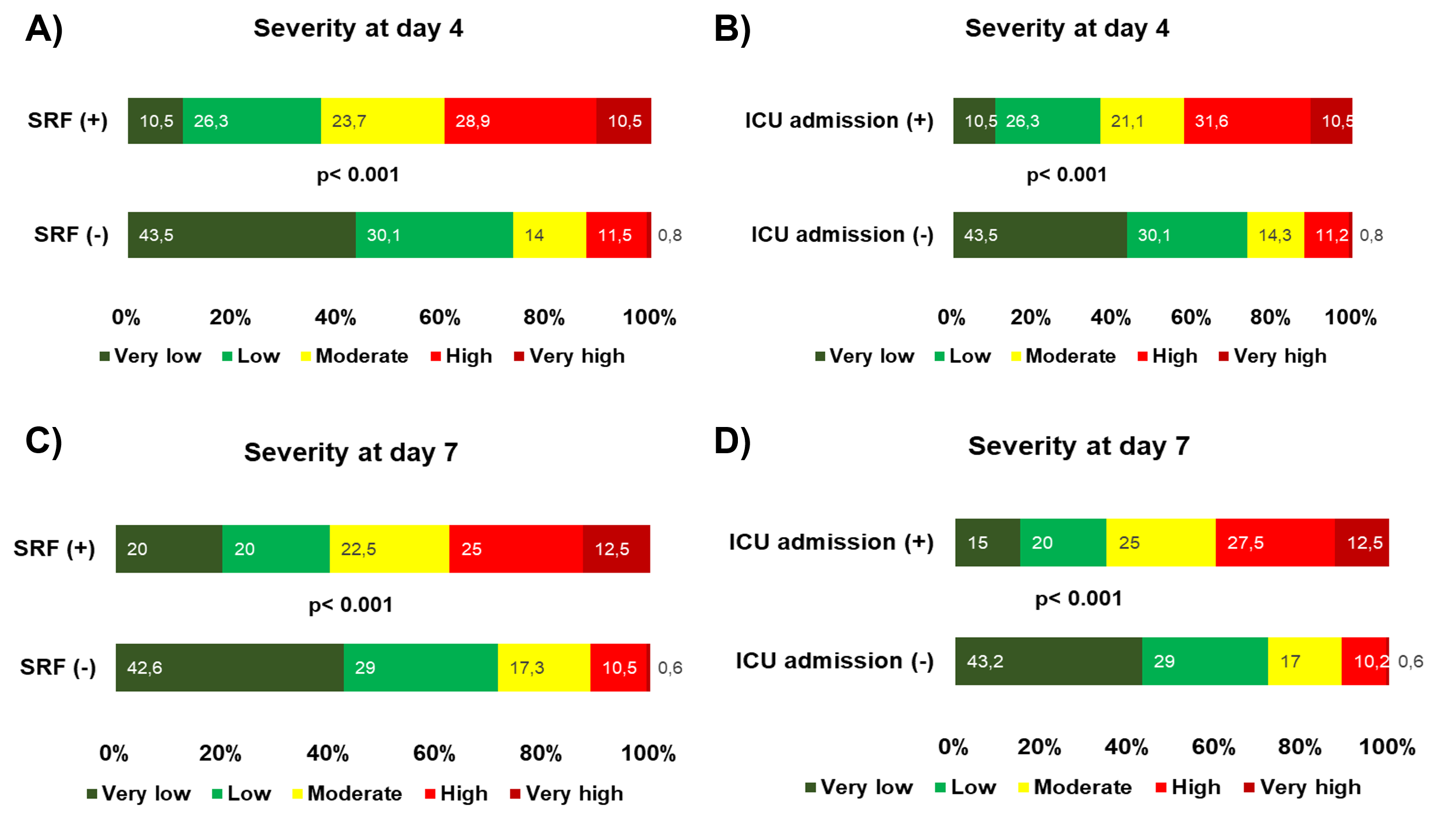


**References**

1. Mitterberger M, Pinggera GM, Colleselli D, Bartsch G, Strasser H, Steppan I, et al. Acute pyelonephritis: comparison of diagnosis with computed tomography and contrast-enhanced ultrasonography. *BJU Int* 2008; 101: 341.

2. Pinson AG, Philbrick JT, Lindbeck GH, Schorling JB. Fever in the clinical diagnosis of acute pyelonephritis. *Am J Emerg Med* 1997; 15: 148.

3. Müller B, Harbarth S, Stolz D, Bingisser R, Mueller C, Leuppi J, et al. Diagnostic and prognostic accuracy of clinical and laboratory parameters in community-acquired pneumonia. *BMC Infect Dis* 2007; 7: 10.

4. Metlay JP, Waterer GW, Long AC, et al. Diagnosis and Treatment of Adults with Community-acquired Pneumonia. An Official Clinical Practice Guideline of the American Thoracic Society and Infectious Diseases Society of America. Am J Respir Crit Care Med 2019; 200: e45-e67.

5. Kalil A, Metersky M, Klompas M, Muscedere J, Sweeney DA, Palmer LB, et al. Management of adults with hospital-acquired and ventilator-associated pneumonia: 2016 clinical practice guidelines by the Infectious Diseases Society of America and the American Thoracic Society. *Clin Infect Dis* 2016; 63: 1.

6. Zilberberg M, Shorr A. Ventilator-associated pneumonia: the clinical pulmonary infection score as a surrogate for diagnostics and outcome. *Clin Infect Dis* 2010; 51: 131.

7. Calandra T, Cohen J. The international sepsis forum consensus conference on definitions of infection in the intensive care unit. *Crit Care Med* 2005; 33: 1538-1548.
